# Acceptability of remotely supervised Home-Based transcranial direct current stimulation combined with Cognitive-behavioural-based app for peripartum depression: perspectives from women with lived experience and mental health professionals

**DOI:** 10.1038/s41598-026-35443-3

**Published:** 2026-02-23

**Authors:** Ana Ganho-Ávila, Andreia Cruz, Nina Szczygiel, Ana Tomás, Catarina Azevedo, Pedro Bastos, Mariana Moura-Ramos

**Affiliations:** 1 Center for Research in Neuropsychology and Cognitive and Behavioral Intervention (CINEICC), Faculty of Psychology and Educational Sciences – University of Coimbra, Coimbra, Portugal; 2 Faculty of Psychology and Educational Sciences – University of Coimbra, Coimbra, Portugal; 3 Research Unit in Governance, Competitiveness and Public Policies, Department of Economics, Management, Industrial Engineering and Tourism, University of Aveiro, Aveiro, Portugal; 4 Clinical Psychology Unit, Unidade local de Saúde de Coimbra, Coimbra, Portugal

**Keywords:** Remotely supervised home-based tDCS, Peripartum depression, Qualitative, Focus groups, Acceptability, Digital interventions, Prefrontal cortex, Psychology, Health services

## Abstract

Peripartum depression (PPD) has a global prevalence of 20% significantly impacting societies. Yet, existing treatments face barriers for its uptake. This study examined the acceptability of a remotely supervised home-based transcranial direct current stimulation (tDCS) solution combined with a CBT-based app (the FLOW Neuroscience solution) for PPD, among women (experts by experience; EEs) and healthcare professionals (HPs). Fifteen EEs and 14 HPs participated in focus groups informed by the Theoretical Framework of Acceptability. Narratives were iteratively refined, using the template analysis method. Participants were overall positive about the treatment considering it supports patients’ autonomy, freedom of choice and a universally accessible perinatal mental health care system. However, they also highlighted several concerns, such as the limitations of exclusively remote/virtual and bot-lead interventions which, on the one hand, may address fear of stigma about perinatal mental health, but on the other may also increase loneliness in depressed patients. Additionally, participants emphasized the need for effective communication strategies to build trust over health innovation and recommended the integration of home-based remotely supervised tDCS treatments combined with a CBT-based app into existing care models. Our findings suggest that while the FLOW solution addresses existing gaps in PPD it also presents some limitations. Here we provide user-centered actionable insights into the adjustment of this innovative, home-based solution to enhance access and engagement in PPD care.

**Supplementary Information:**

The online version contains supplementary material available at 10.1038/s41598-026-35443-3.

^*^ganhoavila@fpce.uc.pt.

## Introduction

Peripartum depression (PPD) is a non-psychotic depressive episode occurring during pregnancy and/or within the first 12 months postpartum that negatively affects the woman, the fetus/newborn, their family and society^1–3^. Albeit context-specific, recent estimates on PPD suggest a global prevalence of 17.4%^4^.

Clinical decision-making in PPD should consider individual factors, case-by-case accessibility barriers and leverages to treatment, and user’s attitudes, values and preferences, significantly influencing treatment adherence and clinical outcomes^5,6^.

In PPD, pharmacotherapy and cognitive-behavioral therapy (CBT) are first-line treatments^7^, both presenting limitations. Medication-based treatments are most often offered to women due to their high accessibility to clinicians and patients in general as a standard treatment, despite the lack of data on their efficacy during pregnancy and in the postpartum^8^. However, only 23–50% of pregnant women accept antidepressants during pregnancy and more than 40% that use antidepressants before pregnancy interrupt their use^9^, due to apprehension towards antidepressants in the peripartum period^10^. CBT presents moderate efficacy as a stand-alone intervention, but it is not universally accessible^11^.

Transcranial direct current stimulation (tDCS) seems an interesting alternative to manage depressive symptoms in the peripartum period^12^. tDCS is a brain stimulation technique that delivers constant, low-intensity (1–4 mA), direct current to cortical areas, modulating the excitability of the neuronal membranes underneath^13^. As of today, one randomized controlled trial^14^, one single arm^15^, and one case study^16^ were published on the efficacy of tDCS to manage PPD during pregnancy. Across the three studies, tDCS showed consistent moderate clinical improvement with p-values ranging from 0.01 to 0.05, both pre-to post treatment and between active treatment and sham. Only mild, transient maternal adverse events and only mild, transient maternal adverse events. No fetal adverse effects were reported, aside from one preterm birth whose direct association with the treatment was not confirmed. Otherwise, follow-up periods ranging from four weeks to three years postpartum, confirmed the absence of other complications. One RCT in pregnant women is currently ongoing (clinicaltrials.org ID: NCT05097586). Additionally, only one case study examining the effect of tDCS in PPD in the postpartum is available^17^ reporting clinical improvement and trivial and expected mild transient effects to the mother (headache and tingling sensation) and no reported adverse events related to the fetus.

Our current knowledge on women’s acceptability of tDCS in the perinatal period is limited to dropout rates, with the literature showing moderate to good retention rates (59.1–80%). Reasons for discontinuation include mostly barriers posed by daily visits for clinic-based treatments^14,15^. Similarly, knowledge about the perspectives and values associated to the use of tDCS in PPD by health professionals and recipients (peripartum women with lived experience of PPD) is limited.

In this study, we aimed to address this gap by conducting the first qualitative study to understand women with lived experience of PPD (experts by experience; EEs) and health professionals’ (HPs) acceptability towards the use of home-based remotely supervised tDCS combined with a CBT app. The definition of acceptability adopted derives from the multi-construct Theoretical Framework of Acceptability (TFA) of healthcare interventions aimed to assess acceptability from the perspective of users^6^. Accordingly, acceptability consists of seven components: the *affective attitude* about the intervention; the *perceived burden*/*effort* required to participate in the intervention; the *opportunity costs* (to what extent individuals consider giving up their benefits/profits/values to engage in the intervention); *ethicality* or alignment the individual’s value system; *intervention’s coherence* (the participant’s understanding of how the intervention works); *perceived effectiveness* (the extent to which the participant understands that the interventions will likely achieve its purpose); and *self-efficacy* (confidence in performing the needed behaviors for the intervention).

The FLOW Neuroscience combines a tDCS Class IIa medical device with a CBT-based mobile application and is approved for the treatment of unipolar major depressive disorder in adults. While it is available in the market freely, it has recently started to be prescribed in the National Health System, in the UK, within a large clinical study. Nevertheless, the efficacy of FLOW is still contradictory^18-20^, despite its high acceptability by patients in an open-label study^21^. However, its efficacy and acceptability has never been tested in pregnant and postpartum women.

## Materials and methods

This study was approved by the Comissão de Ética para a Saúde of the Centro Hospitalar e Universitário de Coimbra, received the reference number OB.SF.055.2022, and was approved on the 02.05.2022 (Ref. 209/CES), complying with the Declaration of Helsinki and all relevant national and institutional guidelines for research involving human participants. Informed consent was obtained from all individual participants included in the study.

### Study design

We used focus groups to evaluate the acceptability of the FLOW Neuroscience solution – a tDCS treatment combining the Flow FL-100 device (delivering 2 mA electric current to the left dorsolateral prefrontal cortex) with a CBT-based mobile application. We have implemented in-person and online focus groups with EEs with diverse clinical trajectories and in-person focus groups with HPs from different academic backgrounds.

A deductive-inductive approach^21^ was employed to assess participants’ perceptions.

### Recruitment and participants

EEs were identified by the local perinatal mental health services and through the snowball method and were recruited by phone between October 2022 and January 2023. Eligibility criteria included being 18 years old or older, fluent in the native language, and to have personal experience of perinatal depression or anxiety disorders. Exclusion criteria included history of previous psychiatric diagnosis outside of major depressive disorder or anxiety, and any current mental health condition. HPs working at the local perinatal mental health services were identified by the service administration and recruited between November 2022 and January 2023. Inclusion criteria included being native-speaking healthcare professionals currently working in maternal health services. Participants were contacted by via email or phone to provide detailed information about the study, obtain informed consent, and coordinate the meeting schedules.

### Data collection

Four in-person (at the research center) and one online group via Zoom (Zoom Video Communications, Inc., 2022) sessions were conducted with EEs and three heterogeneous in-person groups were conducted with HPs at the maternal mental health services facilities. EE groups’ heterogeneity considered diversity in age, marital status, number of children, and previous diagnosis. HPs groups’ heterogeneity considered background diversity (psychologists, psychiatrists, obstetricians/gynaecologists, nurses, primary care and secondary care HPs) and seniority diversity (from residents to senior specialists).

Two semi-structured interview guides were prepared, deducted from the TFA (Sekhon et al., 2017), by two senior researchers in perinatal mental health and TFA (AGA and MMR) and one junior researcher (AT), in collaboration with EEs and HPs. For EEs, the interview topics included: ​​1) Individual impressions about the combined treatment; 2) Factors that lead women to choose this treatment; 3) From whom women prefer to receive information about the treatment; 4) When do women prefer to receive information about the treatment; 5) Preferred context to perform tDCS sessions; 6) Perceived characteristics of the women that most likely accept tDCS. For HPs, the interview topics included: (1) Individual impressions about the combined treatment; (2) Factors that lead women to choose this treatment; (3) When should HPs offer information about treatments; (4) Where and by whom should information about treatment options be offered; (5) Factors that contribute to the prescription of the treatment.

*Apriori* templates were based on the interview guides and included first-order codes (5 for EEs and 6 for HPs) corresponding to the main themes, 0–8 s-order codes (or subthemes) and 0–3 third order codes. To better align with participants’ narratives, codes were modified - new ones were inserted as needed, eliminated if considered unnecessary and changes in codes hierarchy were performed - throughout data collection, according to identified inadequacies.

Focus groups were facilitated by the senior researcher (moderator, AGA), supported by the junior researcher (observer, AT). Each session started with a brief introduction about the research team, the study, general rules of the focus group and the outline for the session. Before introducing the interview topics, participants were briefly presented to the treatment, including trying the tDCS equipment, downloading the CBT-based app to their smartphones and testing it across the different modules. Focus group sessions lasted on average for 90 min.

### Data management and analysis

Sessions were video/audio recorded for the purpose of verbatim transcription. After transcriptions were completed and anonymised, recordings were eliminated. Participants were identified numerically per group (e.g., P1G1, P2G2). Data was processed using template analysis^21^, with data saturation achieved after five focus groups with EEs and 3 focus groups with HPs^22^. The final template was reached when all relevant units of text were assigned to a code.

Content analysis followed the steps adapted from Graneheim & Lundman^23^: (1) coding meaning units of the text, (2) condensing meaning units (description and interpretation/underlying meaning), (3) assigning meaning units to second-order codes/third -order codes. Codes were further refined during the content analysis. The final template was reached when all relevant units of text were assigned to a code. Frequencies of meaning units were extracted to support the identification of patterns across cases. NVivo version 1.6.1 (QSR International, 2023) was used to support the data analysis process.

Steps 1 and 2 were conducted in pairs (AC/PB and NS/CA) after achieving at least moderate (k ≥ 0.75) inter-rater reliability on a sample of transcriptions. Step 3 was conducted by two researchers independently (AC and NS). Across steps, results were discussed with a third researcher (AGA) and conflicts were resolved collaboratively. Throughout the process, every time the meaning of unit was uncertain, the authors returned to the original transcriptions.

## Results

Thirty-one EEs were contacted, of which 15 accepted to participate. Reasons for not participating included distance and limited transportation to the local area where the focus groups meetings took place (*n* = 10), lack of time / lack of interest (*n* = 2) and current symptoms of depression or anxiety with clinical significance (*n* = 3). Sixteen HPs were contacted of which 14 accepted to participate; two withdrew their participation due to limited availability (Table 1).

**Table 1:** Sociodemographic characteristics.

Experts by Experience (*N* = 15)		Mean / *N*	Min-max / Percentage	
Age		35.07	27–43	
Residence	Urban	9	60.00	
	Semi-urban	1	6.66	
	Rural	5	33.34	
Marital Status	Married or non-marital partnership	5	33.34	
	Single/divorced	10	66.66	
Education	Master’s degree	1	6.66	
	University degree	9	60.00	
	High school	5	33.33	
Number of children	1	14	93.33	
	2	1	6.66	
Current concomitant medication	Yes	12	80.00	
	No	3	20	
Previous diagnosis	PPD	6	40,00	
	PPA	8	53.15	
	Mixed (PPD & PPA)	1	6.66	
BSI		0.60	0.08–1.16	
EPDS		7.80	0–14	
Health Professionals (*N* = 14)		Mean / N	Min-Max / Percentage	
Age		43.64		27–63
Education	University Degree	4		28.57
	Masters	9		64.29
	PhD	1		7.14
Profession	General Practitioner	2		14.3
	Psychiatry	4		28.57
	Ob/Gyn	3		21.43
	Residents	1		7.14
	Community Nurse	1		7.14
	Mental Health Nurse	1		7.14
	Clinical Psychologist	2		14.29
Previous knowledge in NIBS	Yes	6		42.86
	No	8		57.14
Previous knowledge in tDCS	Yes	5		35.71
	No	9		64.29

Note: For ordinal variables, values in the second column correspond to frequencies and in the third column to percentages. For continuous variables, values in the second column correspond to means and in the third column to minimum and maximum values. PPD = Peripartum Depression; PPA = Peripartum Anxiety; BSI = Brief Symptom Inventory; EPDS = Edinburgh Postnatal Depression Scale; NIBS = Non-Invasive Brain Stimulation; tDCS = Transcranial Direct Current Stimulation; Ob/Gyn = Obstetrics and Gynecology; GP = General Practitioner.

**Fig. 1:**
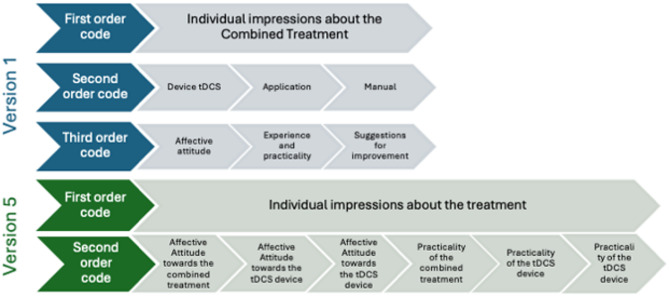
Example of hierarchical changes between codes across EEs template refinement where the third order codes were integrated in the second order codes. The example includes only one first order code – Individual impressions about the combined treatment. Blue: Initial version of the template; Green: Final version of the template.

Major changes produced across templates’ refinement, concerns the elimination of the third order codes by integrating them in second order (see Fig. 1 for an example). The final EEs template included the following second-order codes or themes: (1) Individual impressions about the treatment (including the affective attitude and practicality of the tDCS device, the CBT-based App and their combination); (2) Factors influencing the choice of treatment by EEs; (3) EEs’ preferences for receiving information about the treatment; and (4) Suggestions for improvement. The final HPs template included the following higher-order themes: (1) individual impressions about the treatment (including the affective attitude and practicality of the tDCS device, the CBT-based App and their combination); (2) Factors influencing patients’ choice of the treatment; (3) When, where and from whom should women receive information about the treatment; (4) Factors contributing to the prescription of the treatment; and (5) Suggestions for improvement. For a glance to versions 1 and 5 of the templates including first, second and third order codes, pre- and post-analysis, see Supplemental Files.

Frequencies of codes were extracted from participants’ narratives, supporting the identification of patterns across cases. Frequencies correspond to the number of meaning units per second-order code (e.g. “2/4” correspond to 2 entries of one meaning unit out of the total entries among all meaning units within one second-order code). For the full list of first-order codes, second-order codes and meaning units, see Figure S1 on Supplemental Files.

### Experts by experience (EEs)

#### Individual impressions about the treatment

EEs affective attitude about the treatment were mainly positive (74%). The combination of tDCS with an app captured EEs interest in its innovative nature (2/4) and enthusiasm (1/4) and, to a lesser degree, apprehension (1/4). EEs were mostly enthusiastic about the device (6/8) but feared its safety and potential misuse (2/8).

*Yes*,* just the fact that it is non-invasive is super interesting* (P1G5).

The affective attitude towards the app was mostly positive (73%) with EEs being pleased (4/11) and drawn to the app (3/11). However, there were also feelings of disappointment with the lack of human interaction (1/11) and embarrassment with the language (1/11).

*Some questions that make us feel embarrassed or less comfortable* (P1G3).

In terms of practicality, the combined treatment was perceived positively (92%), considering the advantages offered for clinical monitoring and treatment compliance (2/12), its usefulness and suitability (3/12), simplicity (1/12), convenience of a home-based treatment (2/12) and safety (2/12). However, EEs were apprehensive about the limits of exclusively bot-lead interventions (1/12). The connection of the device with the clinician’s platform for monitoring was appreciated (2/7), considered convenient (1/7) and the device considered simple to use (1/7). However, EEs also mentioned the device apparent fragility (1/7), shared their apprehension when using it (1/7) and fears of being autonomous in administering tDCS (1/7) by themselves.

The experience with the app was generally positive (57%) with the app being experienced as advantageous for self-monitoring of symptoms (1/46), simple (3/46), convenient (2/46), highly customizable (7/46), user-friendly (2/46), discreet (2/46), flexible (4/46), suitable (1/46), informative (2/46), and useful (2/46). For some the language was not suitable (7/47). Simultaneously, the app’s inability to replace face-to-face interactions (8/47), the limits on its customization (1/47), the time of CBT sessions (30 min; 3/47) and the expected progressive loss of adhesion by users (1/47) were issues raised.

*I believe that those dealing with anxiety and emotions need someone*,* face-to-face* (P1G3).

### Factors influencing the choice of treatment

For EEs, the safety profile, namely short- and long-term adverse effects of the treatment, both on the fetus and the mother, during pregnancy and breastfeeding, was the factor to start treatment with the highest weight (22%).

*We think that we will harm them*,* that something will pass into the milk*,* but it actually doesn’t have that side effect*,* so it ends up being better* (P2G4).

EEs’ perception about treatment efficacy depended on having access to reliable information about the treatment (18%) from HPs (7/14), scientific literature (6/14), or real-life testimonials (1/14), increasing the likelihood of choosing the treatment, and building trust and confidence in the intervention. The alignment with individual values weighted 17.5% on the decision to uptake the treatment, namely as a medication-free (6/13) alternative, the freedom to choose treatments according to individual preferences (2/13), novelty and curiosity (2/13), differentiation (1/13), non-invasiveness (1/13) and the non-stigmatizing nature of the treatment (1/13).

*Because it’s something different and not medication based*,* I would choose that* (P1G1).

Costs and efforts associated with the treatment weighted 22% on the decision to uptake the treatment. The home-based nature was aligned with women’s needs (4/9), extending accessibility to treatments (3/9).

*(…) also the fact that we can control the time we dedicate to the process is a good thing*,* and it helps avoid travel* (P3G3).

The low monetary cost was additionally considered (2/9). In terms of efforts, the convenience of a home-based treatment (4/9) and the potential shorter time to response to treatment (4/9) contributed to the treatment uptake. However, the higher monetary value of this treatment compared to medication (due to lack of coverage by insurances) (1/9) and the increased time required to complete one session vs. taking a daily pill (2/9) were of concern. As of contextual factors, the presence of symptoms impacting daily functioning weighted 8% when choosing this treatment.

EEs acknowledge that each case is unique (2/7), that the treatment could be particularly interesting to technologically savvy (4/7) and younger women (1/7) with some health literacy (1/7).

### Preferences about receiving information about the treatment

Most EEs prefer to receive information from a mental healthcare professional (unspecified (2/23), psychiatrist or psychologist; 6/23), obstetrician (5/23), general practitioner (4/23), nurse (1/23) or HP from the maternity (1/23)) or a tDCS expert (4/23). Information should be delivered before pregnancy (2/30), during pregnancy (11/30), before symptoms onset (6/30), at early stages of disease (5/30), at any time (4/30), or depending on the woman’s individual situation (2/30).

*After childbirth*,* a woman is 100% focused on the baby. Her attention is largely directed towards the baby*,* which is why*,* in my opinion*,* it’s important to provide information beforehand rather than afterward* (P1G1).

### Suggestions for improvement

A total of 61 entries were suggestions, most of them about the app. These included content topics (6/61) such as customization to the perinatal population (3/6) and personalization to individual needs (3/6). Suggestions concerning the structure (5/61) included the reduction of the initial questioning (3/5), and gamification (1/5). Suggestions for enhanced functionality included having all content available in the app in the mother tongue of the user (2/16), integration of headphones (1/16), face-to-face or online interactions with health professionals (9/16), enabling appointment on-demand (1/16), integration of physical and mental health care (1/16), a forum or community (1/16), and the mandatory usage of the app when switching-on the tDCS device (1/16). Additionally, a broad reaching communication strategy/campaign about the availability of this treatment would be welcome (1/61).

*It would be amazing if it were possible to use headphones and microphones with the helmet and if the sessions were synchronous*,* with the stimulation session happening simultaneously with the app session*,* providing guided sessions on nutrition and relaxation music and similar things* (P3G2).

### Health professionals

#### Individual impressions about the treatment

HPs affective attitude towards the combined treatment was mixed (33% positive and 67% negative), with one enthusiastic reference to the advantages of self-administered treatments (1/5), but several others expressing concerns about the limitations of exclusively remote/virtual and bot-lead interventions (4/5).

HPs affective attitudes toward the tDCS device were also mixed, with some appreciating its comfort and appearance (1/3) and the availability of an alternative treatment (1/3), alongside fear (1/3). Most HPs identified positive practical aspects of the device (10/13), such as the convenience of its home-based nature in the context of the limited resources in the public health system (1/13) and the flexibility to be completed at the same time as other activities (2/13). The experienced comfort of the headset (3/13), its resistance (1/13), simplicity (1/13), and safety profile (2/13) were positively weighted. On the other hand, HPs showed apprehensiveness concerning the size of the headset (male HP; 2/13) and the lack of human interactions (1/13).

*(…) it makes things much easier because it allows them [women] to make better use of their time* (P4G2).

HPs’ affective attitude towards the app was mostly positive (69%) with many being drawn to it (4/13), pleased (4/13) and enthusiastic (1/13), referring to how it fostered interactions that felt close to natural, and within an aesthetically pleasing and user-friendly interface. However, for some the style was too condescending (2/13). Additionally, there was some apprehension about the limitations of bot-lead interventions (2/13).

HP’s feedback about the app’s practicability included positive (41%) and negative (59%) references. The autonomy promoted by the app, its simple and flexible use, and the clinician’s dashboard for clinical monitoring were considered convenient (5/13). The topics embedded in the CBT sessions were considered of relevance (5/13), useful (1/13), adjusted (1/13) and its accessibility convenient (1/13). In fact, as a stand-alone intervention, the app was seen as a viable alternative to traditional methods, offering a comprehensive solution (more than the stand-alone tDCS), and a complementary intervention for primary care in PPD, representing by itself a significant advancement in mental health care.

*In terms of application*,* I think it benefits from the fact that you can do it at home*,* whenever you want*,* however you want (…)*(P3G3).

As for negative aspects related to the app, HPs identified algorithm constraints that limit personalization (1/19), potential language barriers (despite the subtitles in psychoeducational videos; 2/19), limited clinical monitorization (3/19), limits to exclusively bot-lead psychological interventions (2/19), the overall absence of human interaction (3/19), and concerns about feelings of loneliness by already depressed patients (1/19). The commonly high dropout rates for digital health interventions (2/19) and concerns about patient’s data protection (5/19) were also raised.

*(…) the visual expression*,* the contact allows us doctors to draw several conclusions about the course of treatment*… (P3G2).

### Factors influencing women’s choice of the treatment

HPs discussed six factors contributing to women’s choice of treatment. Costs represented 29% of the intercessions, safety 27%, needs/values 20%, perception of treatment efficacy 13%, contextual factors 9% and confidence to self-administer the treatment 2%. HPs considered that communication should be offered by HPs (2/7) and disseminated by influencers/testimonials (1/7) and it strongly influences women’s perception of treatment efficacy and safety, and consequently their choice (7/7).

*It has to be introduced by a professional (…) this gives credibility*,* gives a sense of security…* (P3G2).

Women’s perception about its safety profile and side effects for the fetus/newborn (6/15) or both the fetus and mothers (9/15) is highly valued, addressing the concerns of those women reluctant to medication.

*If there is this possibility*,* and it is non-pharmacological*,* if it is a way of overcoming it without medication*,* perhaps it is much more acceptable* (P3G3).

Contextual factors influencing the adoption of the treatment by women included family support (3/5), women’s insight about their clinical condition (1/5) and acceptance of their condition (1/5). In terms of costs, HPs considered that women would value treatment’s accessibility (9/16), low monetary cost (2/16), and the potential short time to respond to treatment (1/16).

*(…) many people miss appointments*,* even at the hospital level*,* because they are unable to go to the hospital* (P2G3).

However, the high levels of commitment required for self-delivered treatments (2/16; when compared to traditional medication), could lead to dropouts, supporting the need to implement strategies to ensure consistent treatment schedules at home. For some women the home-based nature with reduced human interactions (2/16) were pointed as extra effort decreasing the chances of choosing this treatment. In that sense, this treatment might be in contrast with some women’s needs of human interaction (2/11). On the other hand, managing therapy privately was seen in line with women’s fears of stigma (4/11) and their need to feel free to choose (3/11), contributing to the treatment uptake. Similarly, while those women reluctant to medication would most probably accept the treatment (1/11), misconceptions about non-invasive brain stimulation treatments can affect acceptance (1/11).

### When, where and from whom should women receive information about the treatment

According to HPs, women should be informed about PPD treatments before being pregnant / during pregnancy (6/10), or after childbirth (4/10), and treatment options should be discussed after symptoms’ onset (10/14), although before symptoms onset was also considered acceptable as a preventive action (4/14). Information should be delivered at the primary (2/5) or secondary (2/5) care services, or both (1/5), by any health care professional (3/16) including nurses (1/16), psychologists (1/16), medical doctor (1/16), GPs (2/16), obstetricians (2/16), psychiatrists (1/16) or any professional as long as they are integrated in a maternal health team and are properly trained (5/16).

### Factors contributing to the prescription of the treatment

HPs discussed seven factors contributing to treatment prescription, including the patient’s profile (weighting 56%), values (13.5%), costs (11%), self-confidence to support self-administration of treatment by patients (9.5%), perception of efficacy (4%), efforts (3%), and safety (1.5%). Hence a new third order code arose concerning clinician’s reasoning (1.5%).

Women to whom the treatment would be most beneficial include those reluctant to medication (7/44) or presenting adverse effects to medication (1/44) and those motivated to complete this type of treatment autonomously (4/44), preferring home-based or remote options (4/44), or with low adhesion to other treatments (1/44). The treatment should be prescribed to pregnant (1/44) or breastfeeding (2/44) women, presenting mild to moderate symptoms of anxiety or depression (7/44), and/or risk factors to develop PPD (such as previous mental health conditions; 4/44).

*It depends. For example*,* we have milder symptoms that can be treated with psychotherapy or some other type of treatment*,* or even as part of a strategy for augmenting antidepressants* (P3G1).

Typically, younger women (1/44) with higher educational degree (3/44) and good technological literacy (2/44) would benefit more than women with low education (3/44) or women in need of increased contact with health professionals (3/44). The patient’s personality was also considered (1/44).

The treatment should be considered either as adjunctive treatment when patients present adverse effects to antidepressants, or as first-line option (1/1). HPs showed confidence in educating their patients on the behaviors required for a successful treatment (7/7). Perception of treatment efficacy was consensual (3/3). The information available to HPs about the safety of the treatment (either from scientific literature sources [1/3] or out of experience [1/3]) were identified as factors driving prescription.

The opportunity of extending treatment options (2/9) and broadening accessibility to health care (3/9) were encouraging factors whereas ethical dilemmas about non-human-led interventions, AI-based diagnosis (3/9) and potential undesirable results from indirect monitoring of remotely supervised treatments (1/9) were discouraging. To a smaller degree, the efforts required to deal with stigma around brain stimulation (1/2) and the time required to educate patients (1/2) were considered.

*(…) if the postpartum woman undergoes this treatment at home*,* managing it herself for a total of six weeks*,* there is no guarantee that she won’t be worse by the end of the process. Meanwhile*,* valuable time has been lost* (P2G3).

Finally, HPs’ values concerning patient’s autonomy (4/10), patient’s freedom of choice (5/10) and the ambition for a universal perinatal mental health care program (given the limited capacity of the public health systems [1/10]) were added as driving prescription.

*… if there is a clinical indication*,* be transparent and give the patient the choice…”* (P4G1).

### Suggestions for improvement

**Fig. 2:**
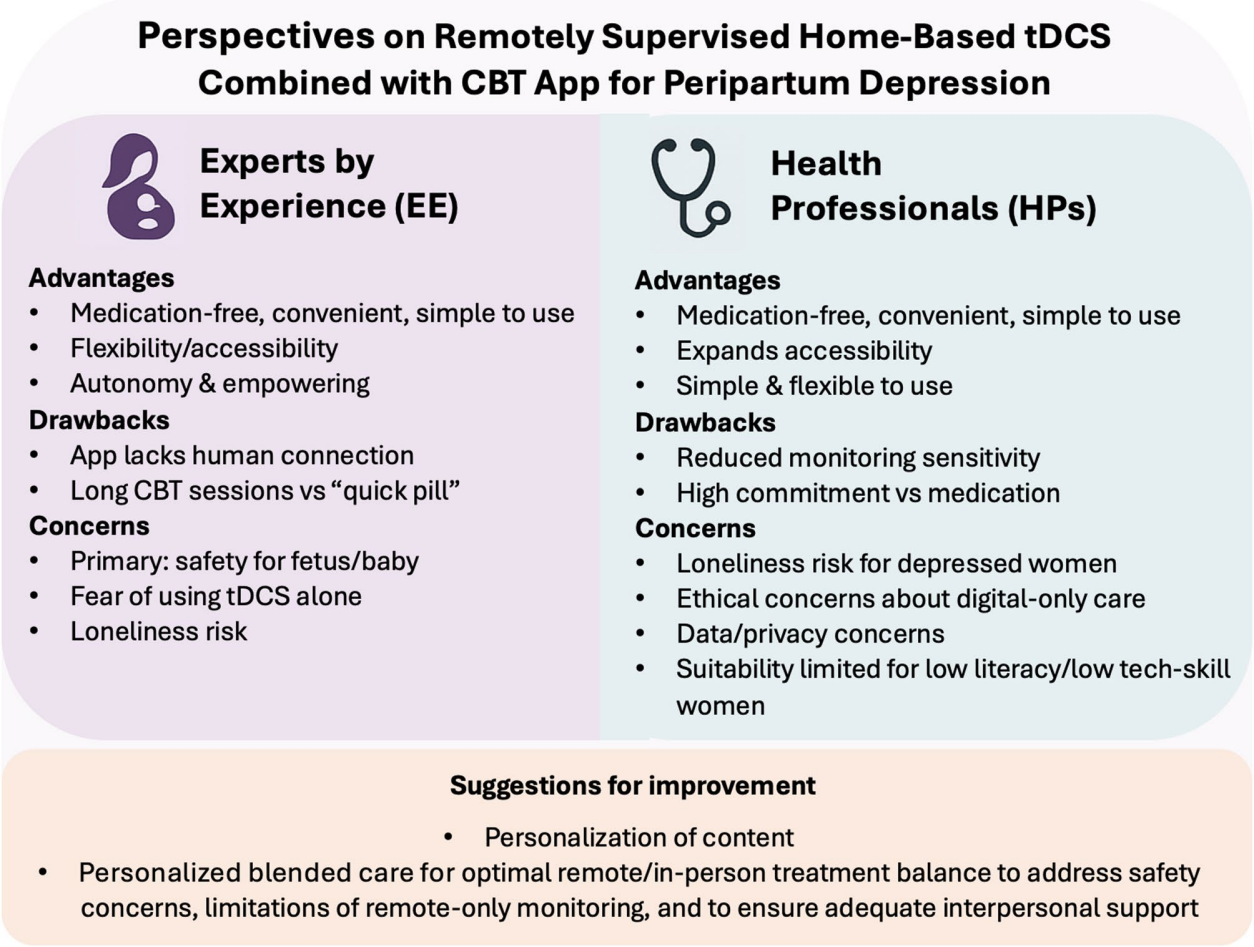
Major takeaways from EEs and HPs evaluations: Advantages, drawbacks and concerns about the FLOW solution.

Several suggestions were offered by HPs, mainly app related. An increased focus of the app content on specific topics related to maternal/perinatal mental health is needed, including clinical and emotional aspects of pregnancy, attachment, postpartum, newborn care, and the negative impact of depression on the fetus/newborn (19/81). Customization features according to distinctive clusters of PPD symptoms, the perinatal period or individual preferences were brought up (12/81). Language adjustments were suggested (4/81) but the degree of formality/directiveness varied according to individual preferences. Structural changes to the app were suggested, including reducing the introductory parts achievable by transferring the introduction to the treatment to an in-person consultation (4/81). Most importantly, HPs agreed on the need to complement self-administered tDCS, self-lead digital intervention and remote monitoring with face-to-face sessions with an HP (32/81). For a synthesis see Table 2; Fig. 2.

**Table 2:** Valence of coded meaning units toward the intervention among EEs and HPs.

	EEs		HPs	
Component	Positive (%)	Negative (%)	Positive (%)	Negative (%)
**Affective attitude toward the combined treatment**	74%	26%	33%	67%
**Affective attitude toward the app**	73%	27%	69%	31%
**Costs and Burden**	67%	33%	NR	NR
**Practicality**	92% (combined treatment)	8%	41% (app)	59%

Note. NR = Not reported. Values represent proportions of coded meaning units and do not correspond to percentages of participants.

## Discussion

In our study, we aimed to assess, for the first time, the acceptability of FLOW for PPD by EEs and HPs, using focus groups and template analysis to deductively and inductively analyze data from the participants’ narratives.

Overall, EEs were more open to the integration of FLOW in the health care whereas HPs were more conservative regarding the limits of completely remote/virtual/self-led treatments, presenting several concerns.

EEs and HPs agreed on several core aspects such as the simplicity and convenience of the treatment, reporting self-confidence to use it autonomously/to educate patients to use it. FLOW seems to be adjusted to both groups in terms of broadening women’s choices for a medication-free alternative and prompting women to self-care while alleviating the health system. EEs and HPs additionally agreed that such a solution would need to be integrated in the existing care through a blended/hybrid care approach that would guarantee at least minimal interactions between women diagnosed with PPD and HPs. Personalizing the ideal combination of remote/virtual treatments and periodic in-person sessions was suggested to address safety concerns, limitations of remote clinical monitoring, and interpersonal needs, while enhancing adherence. Although compliance mechanisms were not explored directly, participants discussed practical and contextual features, such as convenience, perceived safety, and the usefulness of remote monitoring, that they considered essential for sustaining engagement with a predominantly remote intervention.

EEs and HPs were also aligned about when to offer information about maternal mental health and the intervention benefits which can happen already during pregnancy, especially when the first symptoms appear. Younger women, who hold higher education degrees and interested in technology would most likely be the key target group.

To increase engagement, the app should add PPD relevant content and prioritize personalization considering distinctive clinical profiles and perinatal stages. Addressing concerns about data security and integrating additional features, such as requesting medical/clinical appointments through the app, video consultations, or direct messaging, could additionally improve satisfaction and adherence.

Despite the richness of the data collected, this study has its limitations. Our efforts to set heterogeneous focus groups were not entirely successful. The EEs sample presented some homogeneity in terms of the number of children with 14 women reporting one child and only one reporting two. Moreover, this study focused on a specific intervention developed by FLOW, in a specific cultural context and local public health care system (although in both primary and secondary care units), which limits the generalizability of the findings to all home-based tDCS treatments. Further research is needed to explore the acceptability and feasibility of home-based tDCS solutions for PPD and its complementing apps across diverse populations and distinctive healthcare systems. Additionally, in our study clinical outcomes or indicators of symptom improvement were not queried, as the aim of the study was strictly to assess acceptability and not perceived treatment response. However, we consider that future efficacy studies testing remotely supervised home-based tDCS treatments in PPD should integrate patient-reported outcome measures (PROMs) and clinician-reported outcome measures (CROMs) designed for the perinatal context that capture complementary dimensions. While PROMS could document the subjective experience of improvement that women consider meaningful (e.g. emotional regulation, symptom relief, functioning, or capacity to engage in daily caregiving), CROMs, could help track clinically relevant indicators (e.g. symptom trajectories, risk factors, and safety monitoring), providing a more comprehensive and patient-centered understanding of treatment response and informing the refinement of home-based neuromodulation protocols. Furthermore, studies with longer follow-ups assessing acceptability, adherence and clinical outcomes over time could provide a more comprehensive understanding of the intervention’s impact. Finally, although by default, the FLOW solution does not require remotely supervised tDCS sessions, we endorse the use of FLOW exclusively under medical prescription and monitored by properly trained HPs according to the eight rules proposed by Charvet et al.^24^ and the safety recommendations for remotely supervised tDCS (RS-tDCS) outlined by Antal et al.^25^. Together, these frameworks establish the minimum training requirements for clinicians, and staff, define the training skills for patients and define criteria for equipment use and patient monitoring, ensuring the safe implementation of RS-tDCS protocols. Namely, these minimum training criteria for clinicians and support staff includes: (i) the ability to identify and rate potential side-effects; (ii) standardized procedures for monitoring safety before, during, and after each session; (iii) clear criteria for intervention discontinuation; and (iv) communication pathways for rapid follow-up when needed.

The FLOW solution, as a home-based tDCS device combined with a CBT-based app, appears to be a promising option for managing PPD. However, from the perspective of EEs and HPs, addressing identified barriers, particularly safety concerns and the lack of face-to-face interactions, seems crucial for its successful implementation. Users’ suggestions seem aligned with the Digital Therapeutic Alliance framework, suggested by Malouin-Lachance et al.^26^ whereby incorporating relational elements such as empathy and trust, personalization, therapeutic agreement (including treatment goals), as well as ethical concerns should be considered in digitalized treatments to increase acceptability.

By incorporating the perspectives of EEs and HPs, this study aims to offer actionable insights to guide the future development of a user-centered solution combining tDCS and digital health for PPD.

## Supplementary Information

Below is the link to the electronic supplementary material.

Supplementary Material 1

## Data Availability

Raw narrative data from participants are not publicly available in order to protect individuals’ privacy, in accordance with the European General Data Protection Regulation (GDPR). Anonymized data will be made available upon reasonable request addressed to the corresponding author, Ana Ganho-Ávila ( ganhoavila@fpce.uc.pt ).

## References

[1] Bauer, A., Knapp, M. & Parsonage, M. Lifetime costs of perinatal anxiety and depression. *J. Affect. Disord*. **192**, 83–90 (2016). 26707352 10.1016/j.jad.2015.12.005

[2] Fonseca, A. et al. Emerging issues and questions on peripartum depression prevention, diagnosis and treatment: a consensus report from the cost action riseup-PPD. *J. Affect. Disord*. **274**, 167–173 (2020). 32469800 10.1016/j.jad.2020.05.112

[3] Legazpi, P. C. C. et al. Suicidal ideation: prevalence and risk factors during pregnancy. *Midwifery* **106**, 103226 (2022). 34990995 10.1016/j.midw.2021.103226

[4] Wang, Z. et al. Mapping global prevalence of depression among postpartum women. *Transl Psychiatry*. **11**, 543 (2021). 34671011 10.1038/s41398-021-01663-6 PMC8528847

[5] Charlton, R. et al. Selective serotonin reuptake inhibitor prescribing before, during and after pregnancy: a population-based study in six European regions. *BJOG Int. J. Obstet. Gynaecol.* **122** (7), 1010–1020 (2015). 10.1111/1471-0528.13143 25352424

[6] Sekhon, M., Cartwright, M. & Francis, J. J. Acceptability of healthcare interventions: an overview of reviews and development of a theoretical framework. *BMC Health Serv. Res.* **17** (1), 88 (2017). 28126032 10.1186/s12913-017-2031-8 PMC5267473

[7] Nakić Radoš, S. et al. Evidence-based clinical practice guidelines for Prevention, screening and treatment of peripartum depression. *Br. J. Psychiatry* (in press). 227(5):1–12 (2025). 10.1192/bjp.2025.43. 10.1192/bjp.2025.43 PMC12550661 40566968

[8] Brown, R. H. et al. The impact of maternal adverse childhood experiences and prenatal depressive symptoms on foetal attachment: preliminary evidence from expectant mothers across eight middle-income countries. *J. Affect. Disord*. **295**, 612–619 (2021). 34509077 10.1016/j.jad.2021.08.066

[9] Manso-Córdoba, S., Pickering, S., Ortega, M. A., Asúnsolo, Á. & Romero, D. Factors related to seeking help for postpartum depression: A secondary analysis of new York City PRAMS data. *Int. J. Environ. Res. Public. Health*. **17** (24), 9328 (2020). 33322171 10.3390/ijerph17249328 PMC7763494

[10] Hippman, C., Balneaves, L. G., Ryan, D. & Austin, J. Development of the creating comfort in choice theory of decision making regarding antidepressant use in pregnancy: the biggest decision i’ve ever made. *Womens Reprod. Health*. **11** (2), 273–295 (2024).

[11] van Ravesteyn, L., Lambregtse-van den Berg, M., Hoogendijk, W. & Kamperman, A. Interventions to treat mental disorders during pregnancy: A systematic review and multiple treatment meta-analysis. *PLOS ONE*;**12**(3). (2017). 10.1371/journal.pone.0173397. 10.1371/journal.pone.0173397 PMC5373816 28358808

[12] Ganho-Ávila, A., Sobral, M. & van den Berg, M. L. Transcranial magnetic stimulation and transcranial direct current stimulation in reducing depressive symptoms during the peripartum period. *Curr. Opin. Psychiatry*. **37** (5), 337–349 (2024). 38994808 10.1097/YCO.0000000000000954

[13] Antal, A. et al. Low intensity transcranial electric stimulation: Safety, ethical, legal regulatory and application guidelines. *Clin. Neurophysiol. Off J. Int. Fed. Clin. Neurophysiol.* **128** (9), 1774–1809 (2017). 10.1016/j.clinph.2017.06.001 PMC5985830 28709880

[14] Vigod, S. N. et al. Transcranial direct current stimulation (tDCS) for depression in pregnancy: A pilot randomized controlled trial. *Brain Stimulat*. **12** (6), 1475–1483 (2019). 10.1016/j.brs.2019.06.019 31257092

[15] Kurzeck, A. K. et al. Transcranial direct current stimulation (tDCS) for depression during pregnancy: results from an Open-Label pilot study. *Brain Sci.* **11** (7), 947 (2021). 34356180 10.3390/brainsci11070947 PMC8304475

[16] Sreeraj, V. S. et al. Monotherapy with tDCS for treatment of depressive episode during pregnancy: A case report. *Brain Stimulat*. **9** (3), 457–458 (2016). 10.1016/j.brs.2016.03.007 27053386

[17] Laurin, A. et al. Efficacy and safety of transcranial electric stimulation during the perinatal period: A systematic literature review and three case reports. *J. Clin. Med.* **11** (14), 4048 (2022). 35887812 10.3390/jcm11144048 PMC9318834

[18] Borrione, L. et al. Home-Use transcranial direct current stimulation for the treatment of a major depressive episode: A randomized clinical trial. *JAMA Psychiatry*; 81(4):329–337 (2024). 10.1001/jamapsychiatry.2023.4948. 10.1001/jamapsychiatry.2023.4948 PMC10765312 38170541

[19] Woodham, R. D. et al. Home-based transcranial direct current stimulation treatment for major depressive disorder: a fully remote phase 2 randomized sham-controlled trial. *Nat. Med.*; 1–9. (2024). 10.1038/s41591-024-03305-y. 10.1038/s41591-024-03305-y PMC11750699 39433921

[20] Woodham, R. D., Rimmer, R. M., Young, A. H. & Fu, C. H. Y. Adjunctive home-based transcranial direct current stimulation treatment for major depression with real-time remote supervision: an open-label, single-arm feasibility study with long term outcomes. *J. Psychiatr Res.* **153**, 197–205 (2022). 35839661 10.1016/j.jpsychires.2022.07.026

[21] King, N. Using templates in the thematic analysis of text. In: C. Cassell & G. Symon (Eds.), Essential guide to qualitativemethods in organizational research. Sage; 256–270. (2004).

[22] Saunders, B. et al. Saturation in qualitative research: exploring its conceptualization and operationalization. *Qual. Quant.* **52** (4), 1893–1907 (2018). 29937585 10.1007/s11135-017-0574-8 PMC5993836

[23] Graneheim, U. H. & Lundman, B. Qualitative content analysis in nursing research: concepts, procedures and measures to achieve trustworthiness. *Nurse Educ. Today*. **24** (2), 105–112 (2004). 14769454 10.1016/j.nedt.2003.10.001

[24] Charvet, L. E. et al. Remotely supervised transcranial direct current stimulation (tDCS) for clinical trials: guidelines for technology and protocols. *Front. Syst. Neurosci.* **9**, 26 (2015). 25852494 10.3389/fnsys.2015.00026 PMC4362220

[25] Antal, A. et al. Low intensity transcranial electric stimulation: safety, ethical, legal, regulatory and application guidelines (2017–2025: an update). Clin Neurophysiol. ; *(in press).* (2025). 10.1016/j.clinph.2017.06.001 PMC5985830 28709880

[26] Malouin-Lachance, A., Capolupo, J., Laplante, C. & Hudon, A. Does the digital therapeutic alliance exist? Integrative review. *JMIR Ment Health*. **12** (1), e69294 (2025). 39924298 10.2196/69294 PMC11830484

