# Supplementary material for: Acceptability of remotely supervised Home-Based transcranial direct current stimulation combined with Cognitive-behavioural-based app for peripartum depression: perspectives from women with lived experience and mental health professionals
Source: Sci Rep. 2026 Feb 23;16:5140. doi: 10.1038/s41598-026-35443-3 (PMC12929626; doi:10.1038/s41598-026-35443-3)
Supplement: Supplementary file 1 — Supplementary Material 1 [file 41598_2026_35443_MOESM1_ESM.docx]

SUPPLEMENTARY FILES

Scientific Reports

**Acceptability of remotely supervised Home-Based transcranial direct current stimulation combined with Cognitive-behavioural-based app for peripartum depression: perspectives from women with lived experience and mental health professionals**

Ana Ganho-Ávila^a,b^, Andreia Cruz^b^, Nina Szczygiel^b,c^, Ana Tomás^b^, Catarina Azevedo^b^, Pedro Bastos^b^, Mariana Moura-Ramos^a,d^

^a^ Center for Research in Neuropsychology and Cognitive and Behavioral Intervention (CINEICC), Faculty of Psychology and Educational Sciences – University of Coimbra, Coimbra, Portugal

^b^ Faculty of Psychology and Educational Sciences – University of Coimbra, Coimbra, Portugal

^c^ Research Unit in Governance, Competitiveness and Public Policies, Department of Economics, Management, Industrial Engineering and Tourism, University of Aveiro, Aveiro, Portugal

^d^ Clinical Psychology Unit, Unidade local de Saúde de Coimbra, Coimbra, Portugal

**Authors’ Notes**

Correspondence concerning this article should be addressed to Ana Ganho Ávila, Center for Research in Neuropsychology and Cognitive and Behavioral Intervention (CINEICC), University of Coimbra, Coimbra, Portugal

Email: [ganhoavila@fpce.uc.pt](mailto:ganhoavila@fpce.uc.pt)

**Acceptability of remotely supervised Home-Based transcranial direct current stimulation combined with Cognitive-behavioural-based app for peripartum depression: perspectives from women with lived experience and mental health professionals**

SUPPLEMENTARY FILES

- Full versions 1 and 5 of the Templates
- Figure S1. Results of qualitative template analysis. First and second order codes and meaning units extracted for EEs and HPs

THESE TEMPLATES WERE BASED ON THE MULTI-CONSTRUCT THEORETICAL FRAMEWORK OF ACCEPTABILITY (TFA) OF HEALTHCARE INTERVENTIONS (SEKHON’S ET AL., 2017), ACCORDING TO WHICH ACCEPTABILITY CONSISTS OF SEVEN COMPONENTS: AFFECTIVE ATTITUDE, BURDEN, PERCEIVED EFFECTIVENESS, ETHICALITY, INTERVENTION COHERENCE, OPPORTUNITY COSTS AND SELF-EFFICACY.

*********************************************

TYPES OF POSSIBLE CHANGES TO THE ORIGINAL TEMPLATE (King et al 2004)

INSERTION – When relevant text is identified that pertains to the research questions but is not fully covered by any existing code in this template, it is necessary to propose a new code.

ELIMINATION – Removal of a code when, at the end of the transcript review, it is determined that the code is unnecessary.

CHANGING THE HIERARCHY OF CODES – A higher-order code may change its hierarchical position, either upwards or downwards, during the process.

EXPERTS BY EXPERIENCE TEMPLATE (VERSION 1)

1. Individual Impressions about the Combined Treatment (includes duration/number of sessions; sequence of sessions)

a. Device/tDCS

i. Affective attitude (how the participant feels about the device)

ii. Experience and practicality (participant's perception of the device;

understanding of how it works; satisfaction with its use)

iii. Suggestions for improvement

b. Application

i. Affective attitude (how the participant feels about the application)

ii. Experience and practicality (participant's perception of the application; understanding of how it works; satisfaction with its use)

iii. Suggestions for improvement

c. Manual

i. Affective attitude (how the participant feels about the manual)

ii. Experience and practicality (participant's perception; understanding of the manual; missing or excessive information; satisfaction with its use)

iii. Suggestions for improvement

2. Factors Influencing Adherence to the Treatment

a. Perception of treatment efficacy (includes scientific data and access to testimonials)

b. Perceived self-confidence to perform the behaviors required for the treatment

c. Side effects/adverse effects

d. Contextual factors

i. Work conditions

ii. Clinical conditions

iii. Family conditions

e. Economic value (includes costs of adherence/non-adherence, including accessibility)

f. Opportunity costs (extent to which the participant must give up benefits, advantages, or values to adhere to the treatment)

g. Perceived effort (amount of effort the treatment is perceived to require)

h. Alignment with personal value systems

3. Who Would They Like to Receive Information About the Treatment From?

a. Family/friend/acquaintance (includes any relative, friend, or acquaintance from their close or extended social network, whether real or virtual)

b. Professional (includes any professional proficient in the technique, family doctor, perinatal psychiatrist or psychologist, nurse, etc.)

c. Health unit and service (includes health center/hospital/maternity; service/department; public or private)

4. At What Stage of Life Would They Like to Receive Information About the Treatment?

a. At which stage of the perinatal period (e.g., pre-pregnancy, pregnancy, postpartum)

b. At which stage relative to symptoms (before any symptoms are present; only after the first symptoms are present; only when other classical treatments fail...)

5. Perceived Target Population *(Includes which target population is perceived to have the greatest potential for acceptability).*

EXPERTS BY EXPERIENCE TEMPLATE (VERSION 5)

1. Individual Impressions of the treatment (includes duration/number of sessions; sequence of sessions)

1. Affective attitude towards the combined treatment
2. Affective attitude towards the tDCS device
3. Affective attitude towards the App
4. Practicality of the combined treatment
5. Practicality of the tDCS device
6. Practicality of the App

2. Factors Influencing the Choice of Treatment by women

1. Perception of treatment efficacy (includes medical indication, scientific data, and access to testimonials)
2. Perceived self-confidence to perform the behaviors required for the treatment
3. Side effects/adverse effects
4. Contextual factors
5. Economic value (includes costs of adherence/non-adherence and accessibility) and opportunity costs (extent to which the user must give up benefits, advantages, or values to adhere to the treatment)
6. Perceived effort (amount of effort the treatment is perceived to require)
7. Alignment with personal value systems
8. Individual characteristics

3. Women's preferences to receive information about the treatment

1. By whom: Family/friend/acquaintance (includes any relative, friend, or acquaintance from their close or extended social network, whether real or virtual); health professional (includes any professional proficient in the technique, such as a family doctor, perinatal psychiatrist or psychologist, nurse, etc.)
2. When: before, during or after pregnancy, after giving birth; before, early onset of symptoms

4. Suggestions For Improvement

HEALTH PROFESSIONALS TEMPLATE (VERSION 1)

1. Individual Impressions on the Treatment (includes duration/number of sessions; sequence of sessions)

a. Device/tDCS

i. Affective attitude (how one feels about the device)

ii. Experience and practicality (perception of the device; understanding of how it works; satisfaction with its use)

iii. Suggestions for improvement

b. Application

i. Affective attitude (how one feels about the application)

ii. Experience and practicality (perception of the application; understanding of how it works; satisfaction with its use)

iii. Suggestions for improvement (includes content suggestions)

c. Manual and other psychoeducational materials on PPD or combined intervention (includes content and format)

i. Affective attitude (how one feels about the materials)

ii. Experience and practicality (includes perception as a user; understanding of the manual; missing or excessive information, satisfaction with its use)

iii. Suggestions for improvement (includes suggestions on informative, pedagogical, and formatting content)

2. Factors Influencing Patients' Choice of This Treatment

- 1. Perception of treatment efficacy (medical indication, scientific data, and access to testimonials)
  2. Perceived self-confidence to perform the behaviours required for the treatment
  3. Side effects/adverse effects
  4. Contextual factors
     1. Work conditions
     2. Clinical conditions
     3. Family conditions
  5. Economic value (includes costs of adherence/non-adherence and accessibility)
  6. Opportunity costs (extent to which the user has to give up benefits, advantages, or values to adhere to the treatment)
  7. Perceived effort (amount of effort the treatment is perceived to require)
  8. Alignment with personal value systems

3. When in the Life Cycle Should Women Receive Information About the Treatment

1. At what stage of the perinatal period (e.g., pre-pregnancy, pregnancy, postpartum)
2. At what stage relative to symptoms (before any symptoms are present; only after the first symptoms are present, only when other classical treatments fail...)

4. Availability of Treatment Information to Patients

1. Where (e.g., health unit and service, including health centre/hospital/maternity; public or private services/departments)
2. Who (e.g., family doctor, specialist doctor)

5. Factors Contributing to Treatment Prescription

1. Specific patient group (includes information on which target population is perceived as having the highest potential for acceptability)
2. Women’s values and preferences
3. Perception of treatment efficacy (scientific data and access to testimonials)
4. Economic value (includes costs of prescription/non-prescription and accessibility) and opportunity costs (extent to which the healthcare professional must give up benefits, advantages, or values to prescribe the treatment)
5. HPs values and perceived effort (amount of effort perceived in prescribing this treatment)

6. Suggestions For Improvement

TEMPLATE HEALTH PROFESSIONALS (VERSION 5)

1. Individual Impressions of the Treatment (includes duration/number of sessions; sequence of sessions)

1. Affective attitude and practicality of the combined treatment
2. Affective attitude and practicality of the tDCS device
3. Affective attitude and practicality of the App

2. Factors Influencing Patients' Choice of Th Treatment

1. Perception of treatment efficacy (medical indication, scientific data, and access to testimonials)
2. Perceived self-confidence to perform the behaviours required for the treatment
3. Side effects/adverse effects
4. Economic value, opportunity costs and perceived effort
5. Women’s value systems
6. Women’s profile

3. Whom, When and Where Should Women Receive Information About the Treatment

4. Factors Contributing to Treatment Prescription

1. Specific patient group (includes information on which target population is perceived to benefit the most)
2. Women’s values and preferences
3. Perception of treatment efficacy (scientific data and access to testimonials)
4. Economic value, opportunity costs and perceived effort
5. Perceived self-confidence to educate patients on the behaviors required for the treatment
6. HPs values

5. Suggestions for Improvement


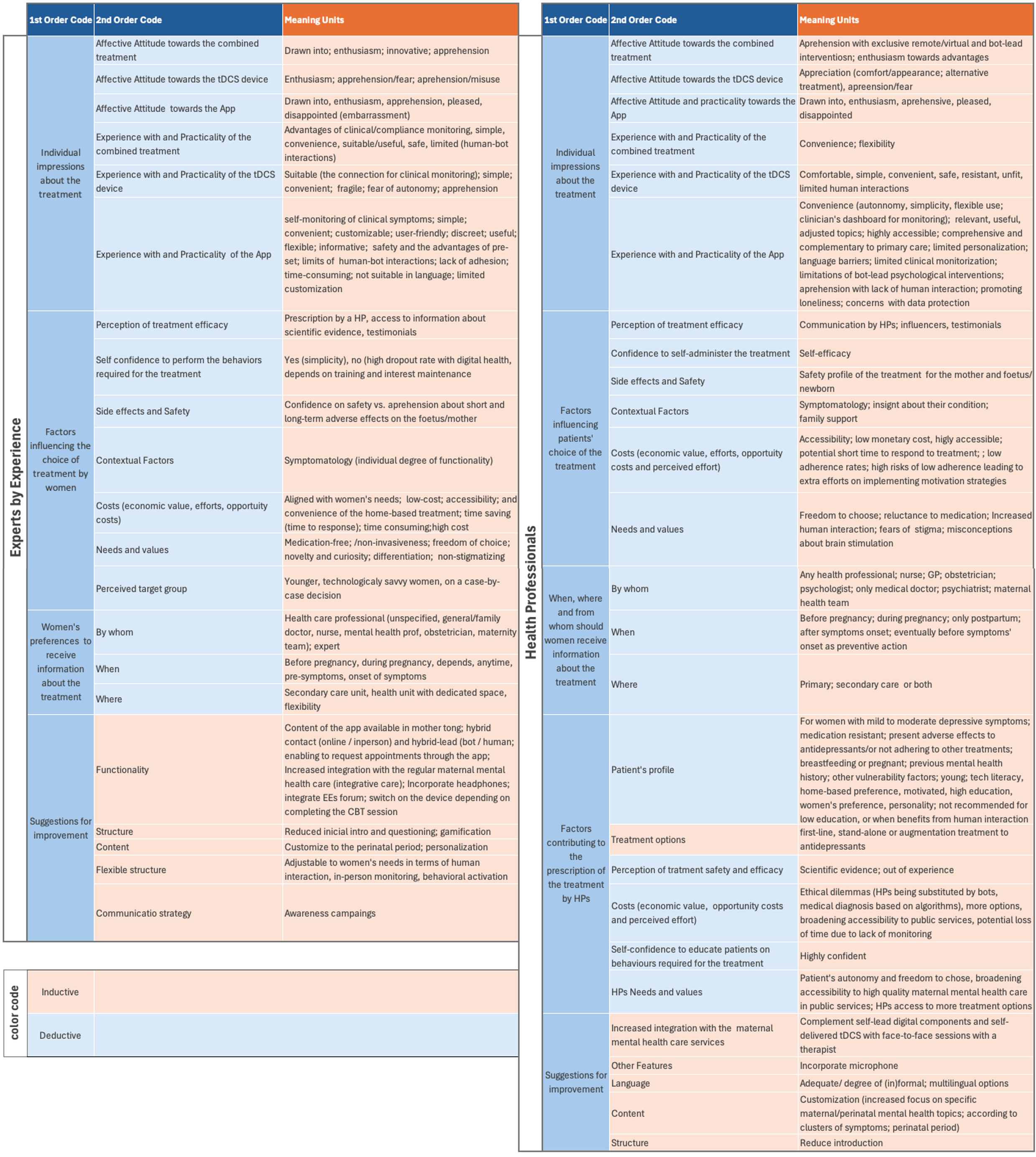


**Figure S1.** Results of qualitative template analysis. First- and second-order codes and meaning units extracted from EEs and HPs narratives. Pinkish background corresponds to inductive codes/units and blueish background to deductive codes/units.
